# The complete mitochondrial DNA sequence of the green algae *Hariotina* sp. F30 (Scenedesmaceae, Sphaeropleales, Chlorophyceae)

**DOI:** 10.1080/23802359.2016.1144089

**Published:** 2016-03-28

**Authors:** Lijuan He, Sulin Lou, Fang Zhang, Shanjun Yang, Chao Zhang, Xiangzhi Lin, Longhe Yang

**Affiliations:** Engineering Research Center of Marine Biological Resource Comprehensive Utilization, Third Institute of Oceanography, State Oceanic Administration, Xiamen, China

**Keywords:** *Hariotina* sp., mitochondrial genome, phylogeny, Scenedesmaceae

## Abstract

The complete mitochondrial genome of the green algae *Hariotina* sp. F30 was obtained in this study using Illumina sequencing data. It is 51 915 bp in length with 36.23% GC content. The genome contains 13 protein-coding genes, 23 tRNA genes and six rRNA genes, all of which are encoded on the heavy strand. AUG is a universal initiation codon among 13 protein-coding genes. UCA is a universal termination codon for most protein-coding gens except UAA in *cox1* and *cob* genes and UGA in *nad6* gene. CUU anticodon for *tRNA-Lys* was detected for the first time in Sphaeropleales.

Regardless the recent mitochondrial genome sequencing boom, it is still largely uncharacterized within green algae Chlorophyceae with only 28 species information published. Scenedesmaceae is one of the largest families in chlorophyceae with 47 genera and 295 species listed in algaebase (Guiry & Guiry 2015). But except the first mitochondrial genome of Scenedesmaceae, *Scenedesmus obliquus* (Kück et al. 2000), this database has not yet been renewed. The mitochondrial genome sequencing of *Hariotina* sp. F30, a freshwater green algae which is characterized by one to three thin elongated connection strands on the top of the cells and mucilage embedding the coenobium (Hegewald et al. 2002), would help us to achieve more knowledge about the mitochondrial genome diversity and evolution within Scenedesmaceae. Considering the poor resolved phylogenetic topology within this family (Johnson et al. 2007; Hegewald et al. 2010, 2013), it would also provide sources to establish a solid phylogeny in further studies.

The whole mitochondrial genome of *Hariotina* sp. F30 which was isolated from Furong Lake in Fujian, China and deposited in Marine Medicinal Organism Germplasm Resources Bank of Third Institute of Oceanography (MMOGRB 0030F) was performed with Illumina HiSeq 2500 platform (Illumina Inc., San Diego, IL). Reads were assembled with SOAPdenovo2 (http://soap.genomics.org.cn). Gene annotation was carried out with Organellar GenoMe Annotator (Wyman et al. 2004) and ORF Finder (Cheng et al. 2013) with *Scenedesmus obliquus* mitochondrial genome as the referee. All tRNA genes were confirmed through tRNAscan-SE and ARWEN search server (Griffiths-Jones et al. 2003; Schattner et al. 2005; Abe et al. 2011).

The mitochondrial circular molecule is of 51 915 bp with 36.23% GC content (accession no. KU145405). The overall nucleotide composition of the heavy strand is 30.62% A, 33.16% T, 19.91% G and 16.32% C. *Scenedesmus obliquus* mitochondrial genome represents an intermediate stage in green algae (Nedelcu et al. 2000). The similar gene content (23 tRNA genes, 13 protein-coding genes and six rRNA) and fragmentation pattern of rRNA genes (four fragments of rnl and two fragments of rns) are also present in our strain, which indicates a typical mitochondrial pattern of Scenedesmaceae. All genes are encoded on the heavy strand. Most of them maintain their integrity except that six introns are found in cox1 gene and one in cob gene. All 13 protein-coding genes have typical AUG initiation codon. Unlike the codon usage in *S. obliquus* mitochondrial genome, apart from the termination codon UCA in most protein-coding genes, UAA termination codon was found in *cox1* and *cob* genes besides UGA in *nad6* gene. The 23 tRNA genes range in size from 71 bp to 87 bp and all can be folded into the typical cloverleaf structure. *tRNA-Thr* was also missing as in *S. obliquus*. The *tRNA-Lys* gene with a CUU anticodon was detected for the first time in Sphaeropleales.

Maximum-likelihood analyses of *Hariotina* sp. F30 and other published mitochondrial genomes in Sphaeropleales (Chlorophyceae) were reconstructed based on the amino-acid sequences of all protein-coding genes (Figure 1). *Hariotina* sp. F30 obviously belongs to Scenedesmaceae. Additional mitochondrial genomes in family Scenedesmaceae will be necessary to enhance the resolution and statistical confidence of infer the intra-familiar phylogenetic relationship.

**Figure 1.**
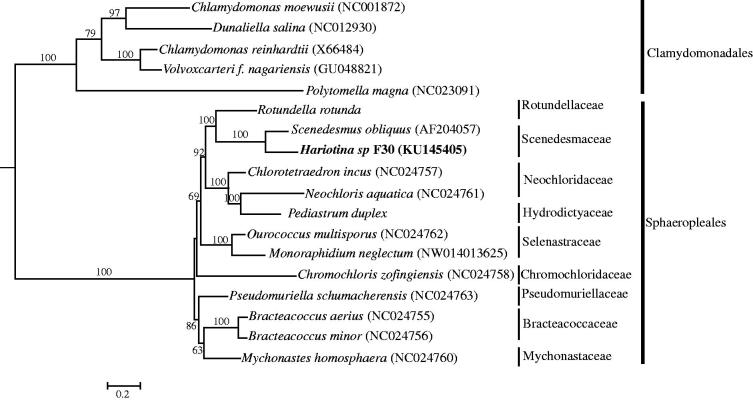
Maximum-likelihood phylogenetic tree of Chlorophyceae based on the amino acid sequences of 13 protein-coding genes. Numbers at each node represent the bootstrap values for ML analysis. Bootstrap values ≤ 60 were deleted. Species of Clamydomonadales were used as outgroups.
